# Delayed onset of ocean acidification in the Gulf of Maine

**DOI:** 10.1038/s41598-024-84537-3

**Published:** 2025-01-15

**Authors:** Joseph A. Stewart, Branwen Williams, Michèle LaVigne, Alan D. Wanamaker, Aaron L. Strong, Brittany Jellison, Nina M. Whitney, Diana L. Thatcher, Laura F. Robinson, Jochen Halfar, Walter Adey

**Affiliations:** 1 School of Earth Sci. University of Bristol, Queens Road, Bristol, BS8 1RJ UK; 2 Kravis Department of Integrated Science, Claremont McKenna College, 888 Columbia Avenue, Claremont, CA 91711 USA; 3 Department of Earth and Oceanographic Science, Bowdoin College, 6800 College Station, Brunswick, ME USA; 4 Department of Earth, Atmosphere, and Climate, Iowa State University, 253 Science, 2237 Osborn Drive, Ames, IA 50010 USA; 5 National Science Foundation, Alexandria, VA 22314 USA; 6 Environmental Studies Program, Hamilton College, 198 College Hill Road, Clinton, NY 13323 USA; 7 Marine and Coastal Science, Western Washington University, 516 High Street, Bellingham, WA 98225 USA; 8 Physical Oceanography Department, Woods Hole Oceanographic Institution, 266 Woods Hole Road, Woods Hole, MA 02543 USA; 9 Department of Environment and Geography, University of York, York, UK; 10 Chemical and Physical Sciences Department, University of Toronto Mississauga, Mississauga, ON Canada; 11 Department of Botany, Smithsonian Institution, National Museum of Natural History, Washington, DC 20013 USA

**Keywords:** Marine chemistry, Climate change, Ocean sciences, Palaeoceanography, Palaeoclimate

## Abstract

The Gulf of Maine holds significant ecological and economic value for fisheries and communities in north-eastern North America. However, there is apprehension regarding its vulnerability to the effects of increasing atmospheric CO_2_. Substantial recent warming and the inflow of low alkalinity waters into the Gulf of Maine have raised concerns about the impact of ocean acidification on resident marine calcifiers (e.g. oysters, clams, mussels). With limited seawater pH records, the natural variability and drivers of pH in this region remain unclear. To address this, we present coastal water pH proxy records using boron isotope (δ^11^B) measurements in long-lived, annually banded, crustose coralline algae (1920–2018 CE). These records indicate seawater pH was low (~ 7.9) for much of the last century. Contrary to expectation, we also find that pH has increased (+ 0.2 pH units) over the past 40 years, despite concurrent rising atmospheric CO_2_. This increase is attributed to an increased input of high alkalinity waters derived from the Gulf Stream. This delayed onset of ocean acidification is cause for concern. Once ocean circulation-driven buffering effects reach their limit, seawater pH decline may occur swiftly. This would profoundly harm shellfisheries and the broader Gulf of Maine ecosystem.

**Supplementary Information:**

The online version contains supplementary material available at 10.1038/s41598-024-84537-3.

## Introduction

Commercial shellfisheries constitute a multi-billion-dollar industry^1^. Consequently, the economic and ecological significance of shellfisheries is acutely felt in coastal communities with abundant populations of calcifying mussels, clams, and oysters, as exemplified by the Gulf of Maine region^2^. Stakeholders, policymakers, and conservationists share a keen interest in safeguarding these regions, especially in the face of rapid changes occurring in our oceans^2,3^.

Currently the hydrography of the Gulf of Maine is influenced by three water masses. Cold, less saline, low alkalinity waters enter the Gulf of Maine from the north in the form of Scotian Shelf Water at the surface (2 °C; 32 psu; alkalinty = 2150 µmol/kg) and Labrador Slope Water at depth (~ 150 m; 4 °C; 34 psu; alkalinty = 2200 µmol/kg)^4–8^. These northern waters mix with warmer (22 °C), more saline (36.5 psu), higher alkalinity (2375 µmol/kg) southern-sourced Warm Slope Waters that derive from the Gulf Stream^4,5^ (Fig. 2). Instrumental observations, paleoclimate proxy records, and model simulations indicate that recent warming of the Gulf of Maine is unprecedented in the last 900 years, and is occurring approximately 3-times faster than the global average (+ 0.03 °C per year over the last 30 years; Fig. 2A;^9–12^). This suggests that the proportion of these water masses may also be changing^6,8,11^. This warming has raised concerns about the thermal stress experienced by commercially relevant shellfish^9,13^. Thermal stress impacts marine calcifiers by inhibiting protein synthesis, metabolism, and larval development, and effects can be further exacerbated under multi-stress, for instance, in combination with lower pH^13^. More than a quarter of anthropogenic carbon emissions have become dissolved into seawater^14^. This addition of dissolved inorganic carbon without modification of seawater alkalinity has led to a decrease in both open ocean surface water pH (− 0.02 pH units per decade; Fig. 2B) and calcium carbonate saturation state^14–16^. Such large magnitude and rapid ocean acidification is also expected to be a key stressor for marine calcifying organisms^17^.

The cumulative impacts of local and global stressors on coastal ecosystems remain poorly constrained; however the nature of “tipping points” and the potential for abrupt ecosystem collapse are of growing concern (review by Trégarot, et al.^18^). Coastal areas experience dynamic spatial and temporal variations in carbonate chemistry due to a range of factors, including tides, biological activity (e.g. primary production), riverine inputs, and circulation. These natural fluctuations overlay anthropogenic carbon invasion into ocean waters adding further complexity to understanding spatial and temporal variability in carbonate chemistry^19^.

**Fig. 1:**
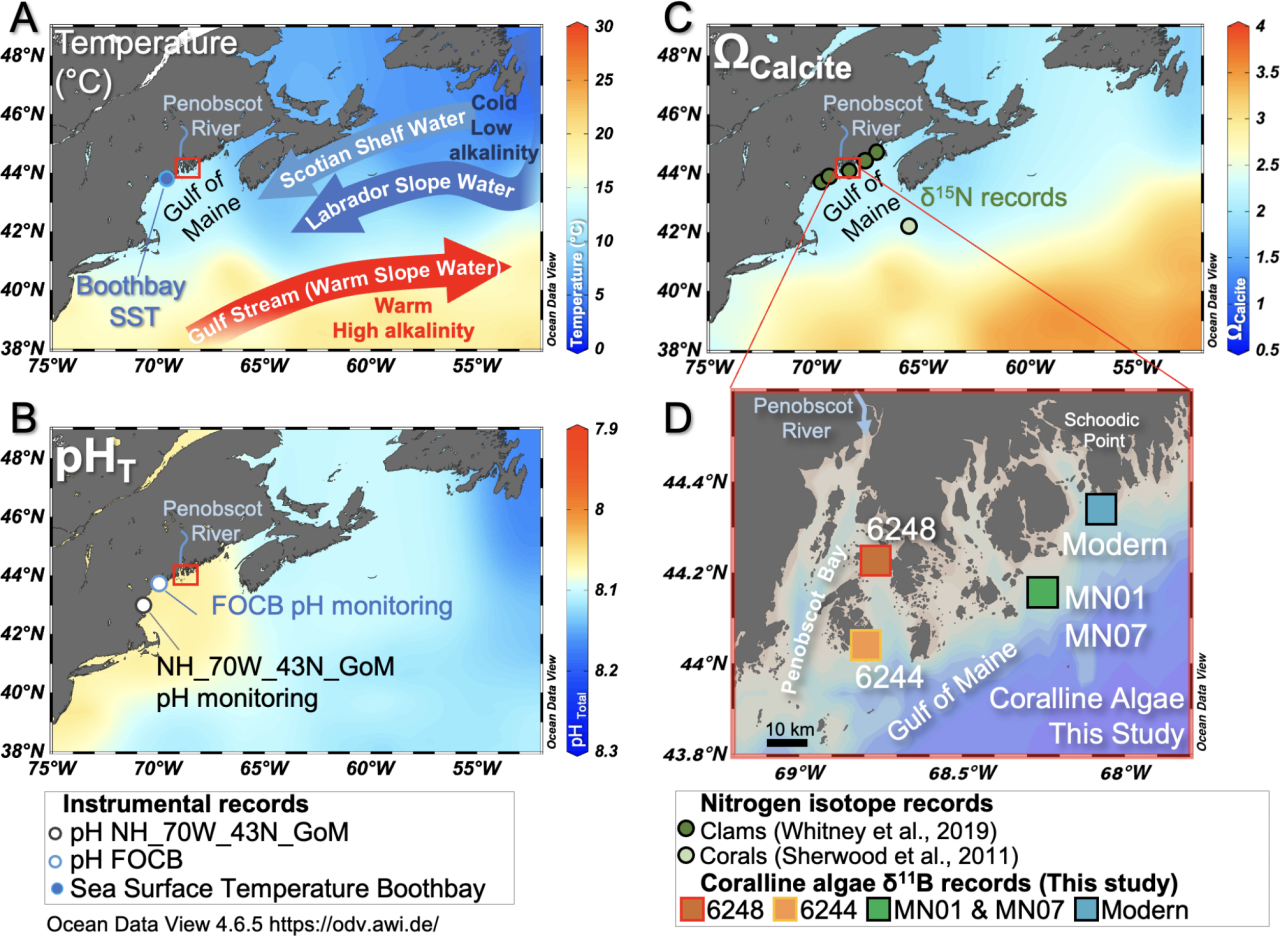
Location of crustose coralline algae samples in this study and modern hydrographic context of the Gulf of Maine. Sea surface temperature (**A**), pH (**B**), and calcite saturation state ((**C**) Ω calculated from bottle measurements of alkalinity and dissolved inorganic carbon) data from Olsen, et al.^4^. Detailed sample locations of the *Clathromorphum compactum* coralline algae specimens used in this study are shown in panel (**D**). The same colour scheme for coralline algae samples is used for symbols in subsequent figures. Locations of proximal sea surface temperature in panel A (Boothbay^10^) and pH in panel B (NH_70W_43N_GoM^26^; FOCB^24^) monitoring stations referred to in the text are shown. Locations of previous nitrogen isotope paleo-proxy records from corals and clams^27,31^ are shown in panel C. Samples for boron isotope measurements of seawater (Table 1) were taken from the same Schoodic Point site as the Modern coralline algae sample (blue square). Base maps drawn using Ocean Data View 4.6.5 https://odv.awi.de/^83^.

In the Gulf of Maine, the effects of anthropogenic carbon influx could be compounded by processes such as organic carbon respiration (which raises dissolved inorganic carbon) or a greater influx of fresher, lower alkalinity waters from rivers and northern-sourced currents^19–23^ (Fig. 1). Conversely, a circulatory switch towards higher alkalinity, saline, Gulf Stream waters from the south could counteract the region’s otherwise low buffering capacity, and could largely offset the effects of anthropogenic CO_2_. However, predicting the trajectory and impacts of coastal ocean acidification in regions like the Gulf of Maine remains challenging, underscoring the need for detailed records of historical pH changes^21^.

**Table 1:** Surface seawater boron isotope measurements from the nearby Schoodic site (44.373°N, 68.077°W).

Site	Collection date (YYYY.MM.DD)	Temperature (Hobo Logger; °C)	Seawater δ^11^B (‰)
Schoodic	2016.05.13	7	39.73
Schoodic	2016.10.05	13	39.77
Schoodic	2017.07.06	9	39.77
Schoodic	2017.11.02	13	39.69
Schoodic	2017.11.30	9	39.69
**Average**			**39.73**
2σ			0.08

Mean average measured value is in bold.

The average measured value is within the uncertainty of open ocean values of 39.61 ± 0.2‰ (2σ) compiled by Foster, et al.^70^.

Recent pH monitoring in the Gulf of Maine reveals increased acidity in coastal waters, with recorded pH values now ~ 7.9 (herein reported on the Total pH scale;^24,25^). This is lower than the typical pH of open ocean waters (pH ~ 8.1;^4^), and there are indications that the coastal waters in this region may be experiencing acidification at a rate exceeding the global average^14^, with a decrease of over 0.01 pH units per year^24^. These instrumental records are, however, very short (only 13 years) and show significant seasonal to inter-annual pH variability in the Gulf of Maine^24,26^, making it impossible to discern whether these low pH conditions are a result of a long-term (decadal) ocean acidification trend, natural coastal factors, or both. Modelling of the Gulf of Maine carbonate system suggests that the steady decline in pH, resulting from atmospheric CO_2_ rise, may have been ongoing since the 1980s^25^. However, the same study found that local rises in temperature and salinity may have partially moderated this decline in pH over the past 30 years^25^. The natural trends preceding the last few decades are not constrained, therefore a new approach is required to assess the natural baseline changes in seawater pH, to determine rates of recent ocean acidification, and to understand the potential drivers of pH in the Gulf of Maine.

Here we present seawater pH records quantitatively derived from boron isotope measurements (δ^11^B; ^11^B/^10^B ratio relative to NIST SRM 951 in ‰) of annually-banded coralline algae (*Clathromorphum compactum*) samples recovered from the Gulf of Maine spanning the last 100 years. These results provide the first insights into multi-decadal surface seawater pH trends in the Gulf of Maine at key coastal locations representative of Maine shellfisheries zones (Penobscot Bay to Schoodic Point, Ellsworth; (Fig. 1)). By extending pH records beyond instrumental observations, we reveal the competing influences of anthropogenic carbon invasion and water mass mixing on coastal water carbonate chemistry.

## Results and discussion

### Historic coastal water pH in the Gulf of Maine

**Fig. 2:**
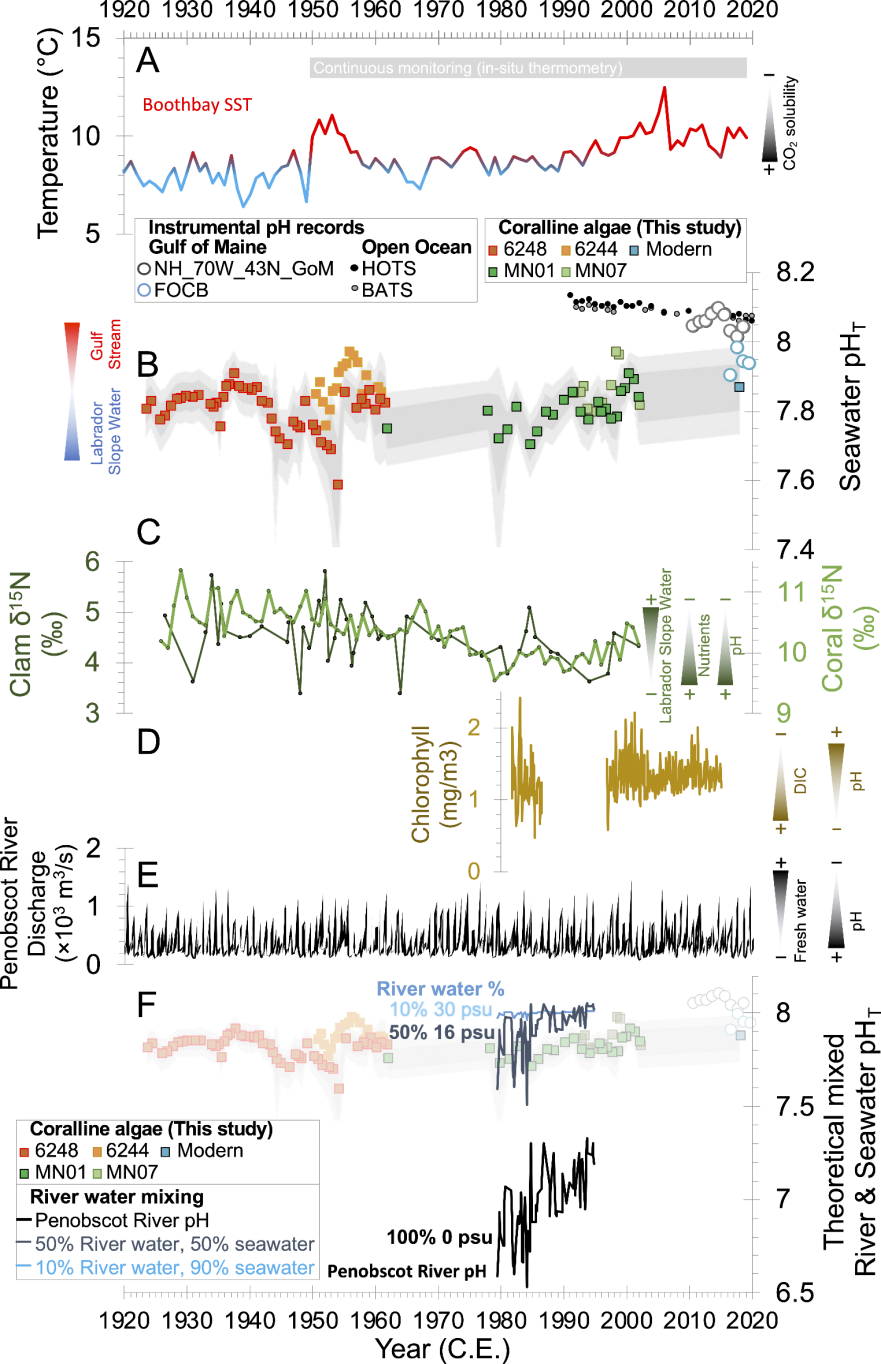
Surface water pH reconstruction and potential drivers. (**A**) Boothbay annual average sea surface temperature^10^. (**B**) Surface water pH reconstructed using coralline algae δ^11^B. Coloured squares refer to layered coralline algae samples 6248 (red), 6244 (orange), MN01 (dark green), and MN07 (light green), and one modern sample (blue). pH estimates are compared to modern instrumental data (circles) from the Gulf of Maine (Casco Bay (FOCB;^24^) and the NH_70W_43N_GoM monitoring station^26^) and open ocean sites Hawaii Ocean Timeseries (HOTS^16^) and Bermuda Atlantic Timeseries (BATS^15^) that document the long term decrease in surface water pH due to increased atmospheric CO_2_ concentrations. Grey error envelopes denote the 68% and 95% confidence intervals on MN01 and 6248 data using the approach of Chalk, et al.^75^. (**C**) Nitrogen isotopic composition of shells^27^ and corals^31^ in the Gulf of Maine region. (**D**) Sea surface chlorophyll concentration (a measure of primary production) in the Gulf of Maine^25,39^. (**E**) Penobscot River discharge measured at^84^ Enfield, Maine^45^. (**F**) Penobscot River pH measured at Eddington, Maine^45^ compared to coralline algae δ^11^B-pH reconstruction. Theoretical mixing of river and coastal seawater assumes fluvial alkalinity of 230 µmol/kg^81^ and dissolved inorganic carbon varying between 261 and 428 µmol/kg to yield the measured river pH values (black line). Riverine carbonate chemistry is mixed in varying proportions with seawater (assumed alkalinity = 2200 µmol/kg, dissolved inorganic carbon content = 2050 µmol/kg, pH = 8.0).

The pH derived from δ^11^B measurements of the most recent coralline algae sample agrees well with modern coastal pH observations from the FOCB site (Fig. 2B). However, the full coralline algae δ^11^B records show that seawater pH at these coastal sites in the Gulf of Maine has remained consistently at pH 7.9 or lower for nearly the entire last century with a quasi-decadal pH variability between 7.7 and 7.9 until the early 1960s (Fig. 2B). This result suggests that the low pH values (pH ~ 7.9) recorded in Casco Bay likely do not signify a long-term decline in surface water pH. Instead, our δ^11^B data imply that coastal pH increased by ~ + 0.2 pH after 1980, reaching values similar to modern seawater by 2000 CE (pH ~ 7.9; Fig. 2B).

Several factors may have contributed to the increase in coastal water pH between 1980 and 2000 CE. However, we posit that the exchange of water masses within the Gulf of Maine, previously demonstrated to induce warming^6,8,11,27,28^, has also shifted in the balance between alkalinity and dissolved inorganic carbon and is the chief cause of the pH elevation. Below we explore the role of water-mass mixing as a driver of pH and suggest a more minor role was played by other factors such as: (i) an increase in seawater temperature, (ii) an increase in surface water primary production, (iii) a decrease in freshwater input via rivers and (iv) an elevation in river pH.

### Increased influence of Gulf Stream derived warm slope waters

Assuming that seawater at the coralline algae sample location (~ 7 m water depth) was broadly in equilibrium with the atmosphere (pCO_2_ = 400 µatm) and attributing the pH variation solely to a modification in seawater alkalinity, a rise of + 0.2 pH units in coastal seawater pH would necessitate an increase of ~ 200 µmol/kg in alkalinity at our study site between the years 1980 and 2020 CE. Therefore, a diminishing influence of the cool, low alkalinity northern-sourced waters and a shift towards predominantly warmer, high alkalinity, Gulf Stream-derived water in the Gulf of Maine in the last 40 years could account for much of the 200 µmol/kg estimated increase in alkalinity with little modification of dissolved inorganic carbon content.

**Fig. 3:**
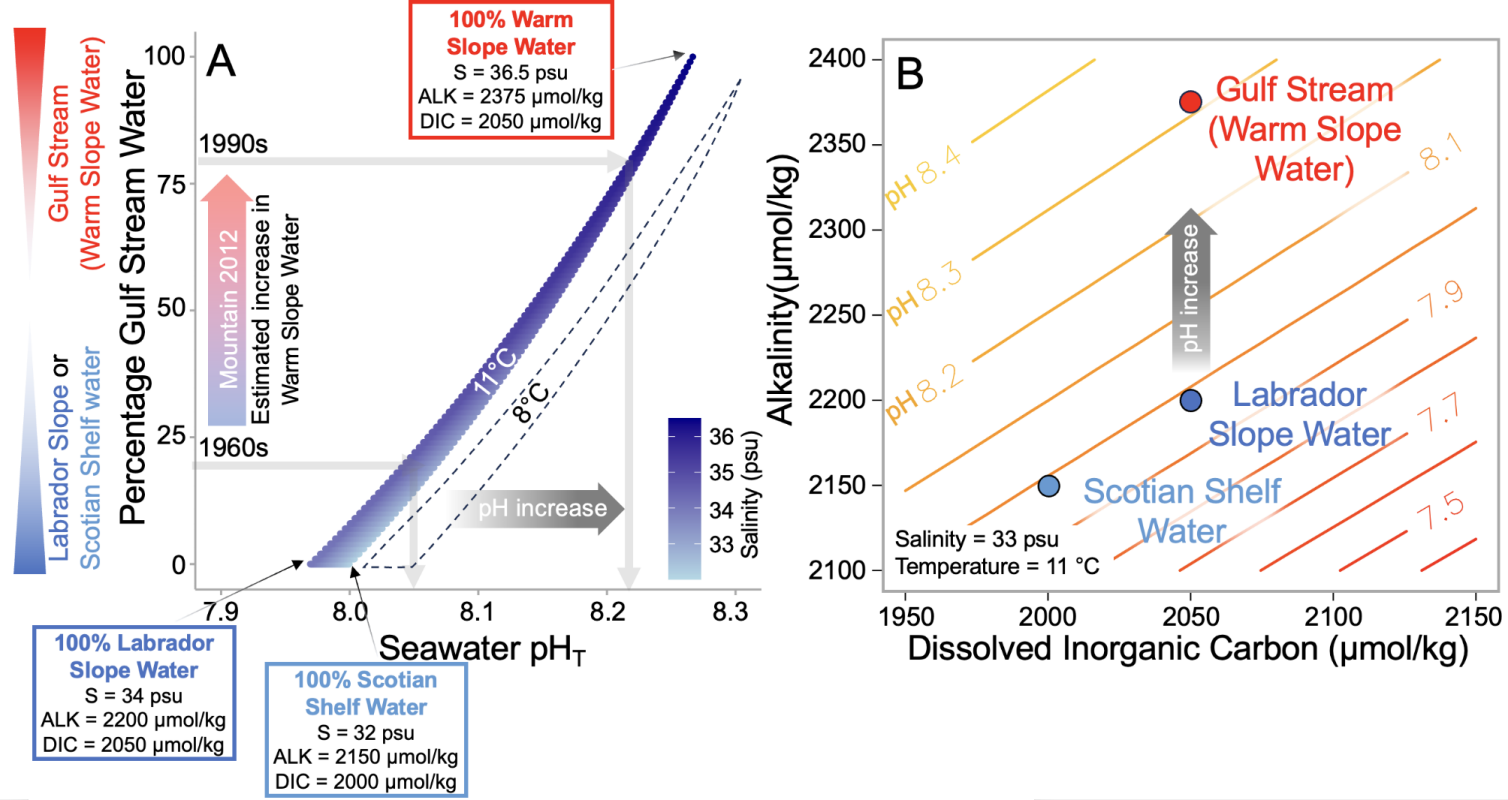
Theoretical pH change due to water mass mixing alone. (**A**) Proportions of the low alkalinity and salinity Labrador Slope Water and Scotian Shelf Water and the high alkalinity and more saline Gulf Stream derived Warm Slope Waters are changed to show the impact on Gulf of Maine surface water pH. The model assumes no riverine mixing component or biological sources/sinks of carbon. Mixing curve is shown for temperature of 11 °C however the dashed area shows the curve at 8 °C (i.e. reflecting recent temperature rise in the Gulf of Maine); however, this temperature effect on pH is largely counteracted by temperature-induced dissolved inorganic carbon release (see Discussion)^25^. The magnitude of Gulf Stream derived Warm Slope Water increase between the 1960s and 1990s estimated from temperature and salinity data in the Gulf of Maine by Mountain (2012)^8^ is shown on the y-axis that translates to a more than 0.15 pH unit increase in pH. (**B**) pH contour plot using fixed temperature and salinity values demonstrating the impact on pH when switching between water masses.

In Fig. 3, we illustrate this effect of a gradual shift from low alkalinity northern-sourced waters to Gulf Stream derived waters using a water mass mixing model. To isolate the impact of water mass exchange, this model excludes any influence from riverine or biological impacts on carbonate chemistry (discussed in detail below). Using compiled temperature and salinity data, a study by Mountain (2012)^8^ suggests that the percentage of Warm Slope Water entering the Gulf of Maine increased from approximately 20% in the 1960s to about 70% in the 1990s at the expense of Labrador Slope Water. More recent temperature and salinity timeseries data further suggest that warm and saline conditions became established in the Gulf of Maine around the year 2010^6^. While local riverine influences or organic carbon cycling may play a role in setting the overall pH value at our coastal study site, this reduced influx of northern, low-alkalinity waters alone would correspond to a more than 0.15 pH unit increase in surface water pH (Fig. 3A).

In addition to the rapid warming (Fig. 2A; Refs.^6,8–10,25^), a diminishing influence of Labrador Slope Water in recent decades is further substantiated by the reduction in dissolved oxygen that has occurred in the Gulf of Maine and nearby Gulf of St. Lawrence since the 1960s; both phenomena are thought to have been exacerbated by an inflow of warmer, lower oxygen, Gulf Stream-derived waters (Figs. 1 and 2A^29,30^). Furthermore, a decrease in δ^15^N nitrogen isotope ratios recorded in clam shells and corals also suggest that a steady change in nutrient regime occurred since the 1960s in the Gulf of Maine (Fig. 2C;^27,31^). In particular, the clam samples used for δ^15^N reconstruction^27^ were obtained from nearby locations to the coralline algae used in our study (Fig. 1). These δ^15^N trends are interpreted as transitioning from predominantly low-nutrient (high δ^15^N) Labrador Slope Waters to high-nutrient (low δ^15^N) Warm Slope Waters derived from the Gulf Stream^5,27,31^.

The precise nature of this phenomenon, whether it signifies a weakening of the Labrador Current or a shift in the position of the Gulf Stream toward the Gulf of Maine, remains uncertain. While the debate over the east versus west basin controls on Atlantic Meridional Overturning Circulation continues to be a topic of significant discussion^32,33^, the Labrador Sea remains a crucial region for deep-water formation of the North Atlantic. Consequently, any changes in the intensity or trajectory of Labrador Slope Waters and/or Gulf Stream demand attention. Such changes could potentially serve as indicators for changes in the buoyancy of high-latitude waters, thereby influencing the long-debated deceleration in Atlantic Meridional Overturning Circulation that carries substantial and far-reaching climatic implications^34,35^.

### Other drivers of coastal seawater pH

#### Temperature rise

The recent pH increase through the last 40 years is concomitant with an approximately 3 °C increase in seawater temperature (from ~ 8 °C to 11 °C) documented at the Boothbay Harbor monitoring station (Fig. 2A; Ref.^10^) and by satellite observations^9^. Without any change to the alkalinity and dissolved inorganic carbon balance, this rise in seawater temperature on its own would alter the dissociation constants for carbonic acid (e.g. pK_1_ from 6.03 to 6.00) resulting in a small lowering of in situ pH (< 0.05 pH unit reduction; Fig. 3A). However, the solubility of CO_2_ in seawater also decreases with temperature, therefore, warming can lead to some dissolved inorganic carbon release from surface waters that can counteract the change due to dissociation constants and causing pH to rise^36^. Previous modelling of the carbonate system suggests that pH in the Gulf of Maine increases by just 0.001 pH units per °C due to CO_2_ release (alkalinity and salinity held constant)^25^. The simultaneous temperature increase of + 3 °C would thus contribute only a modest surface water net increase of 0.003 pH units, falling short of accounting for the full + 0.2 pH shift documented in our record.

#### Primary production

Primary productivity from phytoplankton in the Gulf of Maine is generally high but also varies biannually when episodic nutrient influxes to the surface from slope waters cause blooms in spring and autumn^5,37,38^. These plankton remove dissolved inorganic carbon in surface waters during bloom events. Consequently, where precise carbonate chemistry measurements spanning the last decade are available, seasonal increases in pH broadly align with peaks in chlorophyll concentration, a measure of surface water primary production (Supplementary Fig. 1)^37,39^. The observed rise in seawater pH could therefore result from increased surface water primary production since 1980 CE. However, this hypothesis lacks support from longer-term satellite-based chlorophyll concentration data, which indicate that absolute concentrations remained comparable between the 1980s and the 2000s. Instead, these data suggest that the intensity of primary productivity maxima may have diminished over the past four decades (Fig. 2D; Refs.^25,28,39^). Thus, while there is a scarcity of chlorophyll concentration data for the Gulf of Maine before 1997, making it impossible to rule out local-scale variations in primary productivity, we do not find evidence that primary production drives decadal-scale changes in seawater pH.

During the last century, riverine dissolved organic carbon flux into the Gulf of Maine has generally increased, particularly after the mid-1990s CE^28,40^; Supplementary Fig. 2). The impact of dissolved organic carbon on coastal seawater carbonate chemistry can be complex, as the degradation of these excess organic compounds can add to dissolved inorganic carbon (resulting in lower pH) or release nutrients that stimulate marine primary production, thereby removing dissolved carbon and raising pH^40^. However, the absence of a strong correlation between the timing of the increase in riverine dissolved organic carbon flux, the Gulf of Maine pH-proxy record, and satellite chlorophyll concentration data suggests that dissolved organic carbon has had a minimal impact on coastal seawater pH (Fig. 2B & D; Supplementary Fig. 2).

Macroalgal primary production (rockweed and kelp) also contributes to coastal net primary production in the Gulf of Maine. As such, the macroalgae remove dissolved CO_2_ and can buffer pH in these coastal waters where these coralline algae live^41^. However, in recent decades, growing concerns have emerged regarding the commercial extraction of these macroalgae and its impact on local ecology and seawater carbonate chemistry^42^. Further deterioration of these habitats may hinder the effectiveness of another coastal pH buffering mechanism in the Gulf of Maine, potentially accelerating future pH decline.

#### River water influence

Freshwaters are low in alkalinity; therefore, a decrease in freshwater flux from local rivers draining into the Gulf of Maine could have increased coastal pH (e.g. Refs.^20,43^). However, by comparing our pH record to coastal annual rainfall anomaly data (Supplementary Fig. 2;^44^) and river discharge data from the Penobscot River (Fig. 2E; Ref.^45^) that feeds into the northernmost point of Penobscot Bay (Fig. 1), we find no appreciable decline in either Maine rainfall or Penobscot River discharge during our interval of study. Indeed, riverine runoff data compiled for a state-wide study suggests that precipitation and riverine discharge has increased since the 1980s CE^46^. A decrease in low alkalinity freshwater input via rivers is, therefore, unlikely the cause of coastal pH change.

Historical records of river water carbonate chemistry from the Penobscot River are also sparse. Nevertheless, available continuous data indicate a notable increase in pH, from 6.6 to 7.2, between 1980 and 2000 CE (Fig. 2F; Ref.^45^). By applying a theoretical two-component mixing model (Fig. 2F; see methods) we conclude that this alteration in river pH can only have had a minor effect on coastal seawater pH at our study site. For instance, the addition of 5% river water to Gulf of Maine seawater gives salinity values typical of the coastal waters from which coralline algae were collected (30 to 33 psu), but this would have had an insignificant impact on coastal water pH between 1980 and 2000 CE (~ 0.02 pH unit shift). A more substantial portion of river water (> 50%) would be necessary to induce the 0.2 pH unit shift in coastal seawater pH needed to account for the observed pH increase in our coralline algae reconstruction. However, this would lead to unfeasibly low salinity levels, dropping to less than 19 psu, in which marine coralline algae could not thrive^47^. Consequently, we consider it improbable that river water pH change has had a measurable impact on seawater pH in our study area.

## Conclusions and implications

New pH proxy reconstructions, based on annually-banded coralline algae δ^11^B measurements, reveal that surface seawater pH in the Gulf of Maine has remained consistently low (pH ~ 7.9) for much of the last century. Consequently, these results suggest that the recent, instrumentally-measured, low pH values in the Gulf of Maine do not represent a long-term acidification trend due to anthropogenic carbon emissions. Instead, regional influences on seawater carbonate chemistry have played a dominant role in shaping historical fluctuations in seawater pH within this region.

In particular, the increase in surface water pH between 1980 and 2000 CE is best explained by an increased influence of Gulf Stream derived Warm Slope Waters and a reduction in the cooler, low alkalinity, northern-sourced water masses in the Gulf of Maine. Consequently, our records suggest that the recent direct influence of anthropogenic CO_2_ invasion on seawater pH in the Gulf of Maine has been primarily buffered by changes in water mass mixing. However, this delayed onset of ocean acidification raises significant concerns surrounding ecosystem “tipping points”. The recent dominance of Gulf Stream waters with higher alkalinity suggests that the Gulf of Maine may be close to its maximum buffering capacity. If the influence of the lower-alkalinity Labrador Slope Water were to increase again, or if extreme runoff events became more frequent, rapid acidification could follow, potentially resulting in pH reductions of over 0.1 units. Indeed, temperature and salinity data suggest that even while Gulf Stream waters dominate the slope water mass regime, brief influxes of Scotian Shelf Water remain a feature of the Gulf of Maine at seasonal and interannual timescales (4–7 year cycle)^6^. Such influxes of low alkalinity waters will be felt more acutely in the future as the open ocean pH baseline continues to decline due to ongoing anthropogenic carbon emissions (− 0.02 pH units per decade; Fig. 2B)^14–16^. This could lead to more frequent intervals of extremely low pH and calcium carbonate saturation states, posing serious challenges for marine calcifiers and the fisheries industry they support.

## Methods

### Instrumental records

Published instrumental seawater temperature and pH data are compiled from proximal sites in the Gulf of Maine for comparison to our proxy reconstructions (Figs. 1 and 2). The Boothbay Harbor sea surface temperature record (43.844°N, 69.6417°W;^10^) provides valuable nearshore data for pH calculations (Sect. 4.4) and the assessment of circulation change. The record spans our entire study interval; however, the most reliable temperature measurements were obtained post-1950 CE upon installation of a permanent mooring station at this site^48^.

Seawater pH has been continuously monitored at two sites: the NH_70W_43N_GoM Buoy (~ 12 km offshore; 43.02°N, 70.54°W;^26^) and the nearshore Friends of Casco Bay Continuous Monitoring Station 1 (FOCB) in Yarmouth (43.7519°N, 70.13926°W;^24^). Data collection began in 2010 at NH_70W_43N_GoM and in 2016 at FOCB. pH monitoring data from FOCB have been converted from the NBS to Total pH scale using MATLAB CO2SYS^49^. Although the NBS scale is typically more suitable for freshwater systems, we include these data as they provide the closest continuous pH record to our study site.

To make the data comparable to our coralline algae samples, all pH data – now on the Total scale – have been annually averaged. The NH_70W_43N_GoM buoy, located 12 km offshore, recorded an average pH of 8.06 over the last decade, a value generally representative of open ocean surface waters. The more coastal FOCB site has recorded an average pH of 7.94 since 2016. Differences in pH measurement techniques (such as the use of the NBS scale at FOCB) may explain some of the pH variations between these sites. However, freshwater influences are more likely responsible for the overall lower pH values observed in the nearshore environment at FOCB and at our coralline algae sample sites, compared to open ocean seawater. For this reason, we pay close attention to any concomitant changes in runoff during the interval of study that could impact seawater pH (See Discussion and Fig. 2E,F).

### Coralline algae samples and preparation

Crustose coralline algae are an appealing archive for reconstructing past ocean conditions^50–55^. The taxon *Clathromorphum compactum* is commonly found in mid- to high-latitude coastal waters, exhibits regular annual growth increments^56^, and can have a lifespan of several hundred years^57^. The boron isotopic composition (δ^11^B; ^11^B/^10^B ratio relative to NIST SRM 951 in ‰) of coralline algae has been shown to vary as a function of ambient seawater pH^50,51,53^ with *Clathromorphum* in particular showing significant potential for quantitiatively reconstructing past shallow water pH changes at high latitudes^53,54^.

Identification of crustose coralline algae to the species level can be challenging using morphological traits alone and molecular screening can be helpful^58^. However, it has been extensively demonstrated in the colder North Atlantic and Arctic that modern anatomical/morphological methods are quite successful in making highly reliable identification^47,55,57,59^. The sharply visible, deeply buried intercalary meristem, with a thick overlying photosynthetic epithallium easily identifies members of the genus *Clathromorphum*. DNA analyses show there are only two *Clathromorphum* species in the Gulf of Maine, and *C. compactum* of usable age is easily separated from the alternate *C. circumscriptum* by its color and anatomy^59^.

Both modern and historic *C. compactum* samples were collected from sites in close proximity to each other within the Gulf of Maine (Fig. 1). Samples were collected from hard substrate by SCUBA at a water depth of 10 m (high tide), with a mean depth of 7 m (tidal range > 3 m). The modern sample was collected in 2018 CE from Schoodic Point (44.373°N, 68.077°W). Samples MN01 and MN07 were collected in 2002 and 2003, respectively, from Great Duck Island (44.147°N, 68.253°W). Samples 6244 and 6248 were collected earlier in the year 1962 CE from Arey Cove (44.052°N, 68.791°W) and Eagle Island (44.217°N, 68.768°W) respectively. Samples MN01, MN07, and 6244 were located more than 40 km offshore from the nearest major river input, whereas sample 6248 was situated 27 km offshore from the mouth of the Penobscot River.

Coralline algae were sectioned along the growth axis, polished, and imaged at high resolution. Samples were collected while alive; therefore, the specimen’s uppermost surface corresponds to the year of collection (Supplementary Fig. 3). Preceding years of growth were determined by counting the seasonal cycles in skeletal Mg/Ca values measured along a transect from the surface to the oldest part of the skeleton in each specimen. The Mg/Ca values were measured by laser ablation – optical emission spectrometry at the Keck Science Department of Claremont McKenna College, Pitzer College, and Scripps College, following the methods in Light et al.,^60^ (Supplementary Fig. 3). Together, the larger MN01 and 6248 specimens span the time interval 1923 to 2002 CE.

Specimens were ultrasonicated in Milli-Q water (18.2 MΩ.cm) and air-dried before sampling. The ESI New Wave Micromill was used for precise drilling along growth bands (~ 280 μm thickness and < 500 μm depth into polished sample surface) so that approximately 1 mg of powdered carbonate was recovered per sample. Organic matter was removed by twice treatment in warm 10% H_2_O_2_ (15 min each; 80 °C; buffered in NH_4_OH) ultrasonicating regularly and rinsing thoroughly with boron-free Milli-Q water (18.2 MΩ.cm) between steps. This was followed by a weak acid leach (0.0005 M HNO_3_) before powders were dissolved in distilled 0.5 M HNO_3_^53^.

### Analytical techniques

All analyses were performed at the University of Bristol. An aliquot of the dissolved sample was analysed by ICP-MS using well-characterised, matrix-matched, synthetic standard solutions to give B/Ca ratios used for sample screening (details below). Samples and standards were introduced in 0.5 M HNO_3_. A 0.5 M HNO_3_ and 0.3 M HF acid wash solution was utilised between samples/standards to aid B wash out^61^. Repeat analysis (*n* = 16) of reference materials NIST RM 8301 (Coral), NIST RM 8301 (Foram), and a dissolution of uncleaned JCp-1 (coral powder ) yielded B/Ca values that reproduce to ± 1% RSD with a mean value within 2% of the interlaboratory consensus value for these reference materials^62,63^.

The remainder of the dissolved sample, typically containing 10 ng B, was separated from the carbonate matrix using 20 µl micro-columns containing Amberlite IRA 743 boron-specific anionic exchange resin^64^. The δ^11^B of purified boron samples were measured against NIST SRM 951 by Multi-Collector ICP-MS^64,65^. Samples, blanks, and standard solutions were introduced to the instrument in a 0.5 M HNO_3_ and 0.3 M HF acid matrix again to ensure optimal B wash out^61^. Full procedural uncertainty was assessed using repeat measurement of NIST RM 8301 (Coral) at varying boron concentrations. Reproducibility of this reference material (and samples) was dependent on boron concentration of the solution analysed, for example, measurements with [B] between 4 and 15 ng/g (*n* = 10), 15 to 25 ng/g (*n* = 13), and 25 and 40 ng/g (*n* = 19), gave average δ^11^B values of 24.10 ± 0.42‰, 24.22 ± 0.13‰, and 24.27 ± 0.18‰ (2σ), respectively. These values are within the uncertainty of the interlaboratory consensus value (24.17 ± 0.18‰) for this reference material^62^. Following the protocol of Rae, et al.^65^, we fit a double exponential relationship to long-term NIST RM 8301 (Coral) reproducibility (Eq. 1; Supplementary Fig. 4):

1 $${\text{2}}\sigma {\text{ }} = {\text{ 1}}.{\text{61 }} \times {\text{ e}}^{{\left( { - 0.{\text{22 }} \times {\text{ }}\left[ {\text{B}} \right]} \right)}} + {\text{ }}0.{\text{17 }} \times {\text{ e}}^{{\left( { - 0.{\text{002 }} \times {\text{ }}\left[ {\text{B}} \right]} \right)}}$$

Equation (1) is then used to assess the analytical uncertainty of coralline algae samples based on the boron concentration of each dissolved solution. Total procedural blanks (*n* = 6) were less than 39 ng of boron, and, thus, were typically < 0.2% of the boron loaded onto columns for samples.

### Calculation of seawater pH

The δ^11^B of the borate ion in seawater increases as a function of seawater pH^66^. Marine carbonates incorporate this charged borate species, therefore, their skeletal δ^11^B can be used to reconstruct seawater pH at the time of formation by using the relationship between δ^11^B_borate_ and seawater pH described in Eq. (2)^67,68^:

2 $$\:\text{pH = p}{K}_{\text{B}}^{\text{*}}-\text{log}\left(-\frac{{{ \delta }}^{\text{11}}{\text{B}}_{\text{sw}}-{ \delta }^{\text{11}}{\text{B}}_{\text{borate}}}{{{ \delta }}^{\text{11}}{\text{B}}_{\text{sw}}-{{ \alpha }}_{\text{B}}{ \delta }^{\text{11}}{\text{B}}_{\text{borate}}-{{1000( \alpha }}_{\text{B}}-\text{1)}}\right)$$

where α_B_ (1.0272) is the fractionation factor between boric acid and borate^69^, δ^11^B_sw_ (39.61) is the δ^11^B of seawater^70^ and p*K*_B_^*^ is the dissociation constant of the two boron species (average value 8.82).

Coralline algae calcify within their cell walls from a fluid with pH elevated relative to ambient seawater that results in skeletal δ^11^B values higher than seawater δ^11^B_borate_ values predicted by Eq. (2) ( ~ + 12‰ higher;^50,51,53,71^). While the internal pH of corals and coralline algae vary as a function of external seawater pH, secondary factors have been suggested to play a role in changing internal pH and skeletal δ^11^B^72,73^. For instance low light levels and water flow speed have been observed to lower the internal pH in corals, however these effects were found to be largely absent in coralline algae (*Sporolithon durum*)^72^. While light levels can impact other aspects of skeletal chemistry (e.g. Mg/Ca ratios^74^), calcification in *C. compactum* is shown to continue to progress even under low light conditions^74^ and we do not expect annual average light levels to have changed significantly during the interval of study. However, the degree to which internal pH is elevated above seawater certainly does vary between coralline algae species, therefore species-specific δ^11^B-pH calibrations are required to convert skeletal δ^11^B to external seawater pH;^50,51,53,71^. We therefore convert our coralline algae δ^11^B measurements to pH using the relationship to the seawater δ^11^B_borate_ obtained in a detailed culture study of *C. compactum* by Anagnostou et al.^53^ that included 12 culture treatments of varying temperature and pH (7.2 to 8.2) measured in triplicate:

3 $$\delta ^{{{\text{11}}}} {\text{B }}({\text{2}}\sigma ){\text{ }} = {\text{ 1}}.{\text{46 }}\left( { \pm 0.0{\text{6}}} \right){\text{ }}\delta ^{{{\text{11}}}} {\text{B}}_{{{\text{borate}}}} + {\text{ 6}}.{\text{91 }}\left( { \pm 0.{\text{72}}} \right){\text{ }}({\text{2}}\sigma )$$

We measured δ^11^B of local surface seawater at the nearby Schoodic site (sampling site of modern coralline algae specimen) between 2016 and 2017 (Table 1) using identical techniques to those detailed for carbonate samples in Sect. 4.3. We find that δ^11^B_sw_ varies little seasonally (± 0.08‰; 2σ), and yields an average δ^11^B value within the uncertainty of open ocean values compiled by Foster, et al.^70^. We calculate p*K*_B_^*^ for each year of the record using an average surface seawater salinity of 30.3 psu recorded at the FOCB monitoring station (< 30 m depth) and Boothbay temperature monitoring data (temperature values between 6 and 11 °C;^10^). These same temperature and salinity values, along with a pCO_2_ value of 400 µatm, are used to calculate the required change in alkalinity needed to explain the pH increase over the last 40 years recorded in our δ^11^B records.

Uncertainty on calculated seawater pH estimates is assessed using the Monte Carlo simulation method of Chalk, et al.^75^. This approach propagates multiple sources of uncertainty including: (i) the *C. compactum* calibration above (Eq. (3)), (ii) coralline algae δ^11^B measurements error (Eq. (1)), (iii) δ^11^B of seawater value (± 0.1‰; conservative estimate), (iv) seawater temperature (± 1 °C; conservative estimate), (v) seawater salinity (± 1 psu; conservative estimate) (1σ). The resultant error envelopes are plotted with our pH reconstruction.

The δ^11^B value of 28.7‰ recorded in the most recent coralline algae sample, representing growth in 2018 CE (Fig. 2B), corresponds to a calculated seawater pH value of 7.87 (Fig. 2B). This is in close agreement with modern seawater observations^26^, particularly those at the inshore FOCB site (pH = 7.94;^24^). Because this historic pH record is chiefly composed of two long-lived coralline algae specimens, we cannot fully discount small local-scale changes in pH regime at these two nearby sites (e.g. subtle differences in productivity, currents, or riverine influence). However, where dates overlap between replicate specimens MN07 and 6244 and the longer records from specimens MN01 and 6248, boron isotope values generally agree well (typically within 1‰), particularly within the youngest layers (typically within 0.5‰; Fig. 2B). These shorter records from replicate specimens MN07 and 6244 specimens suggest a similar pattern of pH change compared to the longer MN01 and 6248 specimens with δ^11^B highs (pH 7.9) in years ~ 2000 and ~ 1956 and lows (pH < 7.8) in years ~ 1994 and ~ 1952 CE.

### B/Ca vs. δ^11^B and sample screening

Generating climate records from coralline algal chemistry requires the collection of well-preserved primary carbonate samples free of diagenetic alteration and recrystallization. Consequently, parts of coralline algae visibly altered or impacted by bioerosion were avoided. It can be difficult, however, to visually assess how far chemical alteration extends around these diagenetic structures. Therefore, we provide additional B/Ca data for our powdered samples to screen for secondary calcite precipitation that could mask environmental signals (Supplementary Figs. 5 and 6). These B/Ca ratios strongly correlate with δ^11^B, both within and between coralline algae specimens (Supplementary Fig. 5). Such trends are commonly found in biogenic carbonates that increase saturation state at the site of calcification (e.g. scleractinian corals;^76,77^) and this finding aligns with similar measurements made in coralline algae cultures studies, including *C. compactum*^51,53^.

In this study, however, we observe a number of powdered coralline algae samples that deviate from this expected positive trend, yielding anomalously high B/Ca and low δ^11^B values: specifically, six samples within specimen MN01 (adjacent samples spanning the years 1967 to 1976 CE) and one sample within specimen 6248 (dated to the year 1956 CE). Many of these divergent values are so low in δ^11^B that a seawater pH value cannot be calculated using Eq. 3. These anomalous samples within specimen MN01 are near large worm borings (Supplementary Fig. 6). Prior assessments of serpulid worm carbonate have demonstrated these structures to be enriched in boron (B/Ca ~ 400 µmol/mol) and characterized by low boron isotope ratios (< 20‰) in comparison to coralline algae^78^. The potential incorporation of a small percentage of this secondary carbonate could significantly impact the boron isotope chemistry of coralline algae, rendering it unrepresentative of ambient seawater pH. We exclude these seven sub-samples from pH reconstruction, as they likely signify intervals contaminated by non-primary carbonate sources (Supplementary Figs. 5 and 6). Our results reveal the importance of paired boron concentration data to ensure that boron isotope ratios in coralline algae accurately reflect primary carbonate composition and, by extension, seawater pH.

### Mixing models

#### Water mass mixing

We use a three-component mixing model to demonstrate the impact of changing proportions of the low alkalinity Labrador Slope and Scotian Slope Waters and Gulf Stream derived Warm Slope Waters on surface water pH in Gulf of Maine (Fig. 3). This model assumes no riverine mixing component so as to isolate the impact of these changing water masses only. We take varying proportions of Warm Slope Waters (assumed alkalinity = 2375 µmol/kg, dissolved inorganic carbon = 2050 µmol/kg, salinity = 36.5 psu;^4–8^), Labrador Slope Waters (assumed alkalinity = 2200 µmol/kg, dissolved inorganic carbon = 2050 µmol/kg, salinity = 34 psu;^4–8^) and Scotian Shelf Waters (assumed alkalinity = 2150 µmol/kg, dissolved inorganic carbon = 2000 µmol/kg, salinity = 32 psu;^4–8^) and calculate the in-situ pH for each ALK: DIC ratio and salinity condition (SeaCarb^79^; dissociation constants from Lueker et al.^80^; Fig. 3A). We do this for temperatures of 8 and 11 °C that represent the magnitude of temperature rise between the 1980s and 2000s^10^ (note that much of this pH decline with temperature however would be countered by a decrease in solubility CO_2_ with temperature^25^). We also show the impact of this theoretical change in seawater carbonate chemistry using a pH contour plot, however in this case salinity and temperature remain fixed (Fig. 3B).

#### River water and seawater mixing

Penobscot River pH increased from 6.6 to 7.2, between the years 1980 and 2000 CE (Fig. 2F;^45^). While the cause of this shift in river water pH remains unclear, a similar pH pattern is also seen in data from the St Croix River, which drains into the northern Gulf of Maine (approximately 100 km north of our study site; Supplementary Fig. 2) suggesting that this is a regional change in river pH. We show another two-component mixing model, this time to assess the small impact of changing river pH on coastal seawater pH at our study site. Penobscot River water alkalinity was assumed to be a constant 230 µmol/kg^81^, from which riverine dissolved inorganic carbon content was calculated using the SeaCarb R package (values from 410 to 260 µmol/kg) and historic river water pH measurement data^45,79^. This river water was mixed in varying proportions with seawater (assumed alkalinity = 2200 µmol/kg, dissolved inorganic carbon = 2050 µmol/kg, salinity = 33 psu; typical of offshore waters in the Gulf of Maine^4^; unmixed seawater pH = 8.0) and the mixed coastal water alkalinity, dissolved inorganic carbon, salinity and pH was calculated (SeaCarb^79^; dissociation constants from Lueker et al.^80^ that are suitable for salinity values down to 19 psu). Assuming constant riverine dissolved inorganic carbon and varying alkalinity had an even smaller impact on the modelled coastal seawater pH. This model does not discriminate between riverine carbonate and non-carbonate alkalinity when assessing changing total alkalinity in coastal waters (e.g^82^). However, we note that altering our assumed value of riverine alkalinity (230 µmol/kg) by ± 50% has little effect on the key finding that river water would need to contribute significantly more than 20% to the alkalinity and dissolved inorganic carbon budget of this coastal system for changing river pH to have impacted local seawater values.

## Electronic supplementary material

Below is the link to the electronic supplementary material.

Supplementary Material 1

Supplementary Material 2

## Data Availability

A supplementary information file includes all new boron isotope and B/Ca data from coralline algae samples in this study.
