# Supplementary material for: Delayed onset of ocean acidification in the Gulf of Maine
Source: Sci Rep. 2025 Jan 15;15:2039. doi: 10.1038/s41598-024-84537-3 (PMC11736168; doi:10.1038/s41598-024-84537-3)
Supplement: Supplementary file 2 — Supplementary Material 2 [file 41598_2024_84537_MOESM2_ESM.docx]

Delayed onset of ocean acidification in the Gulf of Maine

**Joseph A. Stewart** ^a*^**, Branwen Williams** ^b^**,** **Michèle LaVigne** ^c^**, Alan D. Wanamaker** ^d,e^**, Aaron L. Strong** ^f^**, Brittany Jellison** ^c^**, Nina M. Whitney** ^g,h^**,** **Diana L. Thatcher** ^d^**,** **Laura F. Robinson** ^a,i^**,** **Jochen Halfar** ^j^**,** **Walter Adey** ^k^

^a^ School of Earth Sci. Univ. of Bristol, Queens Road, Bristol, BS8 1RJ, UK

^b^ Kravis Department of Integrated Science, Claremont McKenna College, 888 Columbia Avenue, Claremont, CA 91711, USA

^c^ Earth and Oceanographic Science, Bowdoin College, 255 Maine Street, Brunswick, Maine, USA

^d^ Geological and Atmospheric Sciences, Iowa State University, 151 Science 2237 Osborn Dr. Ames IA, USA

^e^ National Science Foundation, Alexandria, VA, USA

^f^ Environmental Studies Program, Hamilton College, 198 College Hill Road, Clinton, NY 13323, USA

^g^ Marine and Coastal Science, Western Washington University, 516 High Street, Bellingham, WA, 98225

^h^ Physical Oceanography Department, Woods Hole Oceanographic Institution, 266 Woods Hole Road, Woods Hole, MA, 02543

^i^ Department of Environment and Geography, University of York, York, UK.

^j^ Chemical and Physical Sciences Department, University of Toronto Mississauga, Mississauga, ON, Canada

^k^ Department of Botany, Smithsonian Institution, National Museum of Natural History, Washington, DC, 20013, USA

*correspondence [joseph.stewart@bristol.ac.uk](mailto:joseph.stewart@bristol.ac.uk)

Supplementary Information


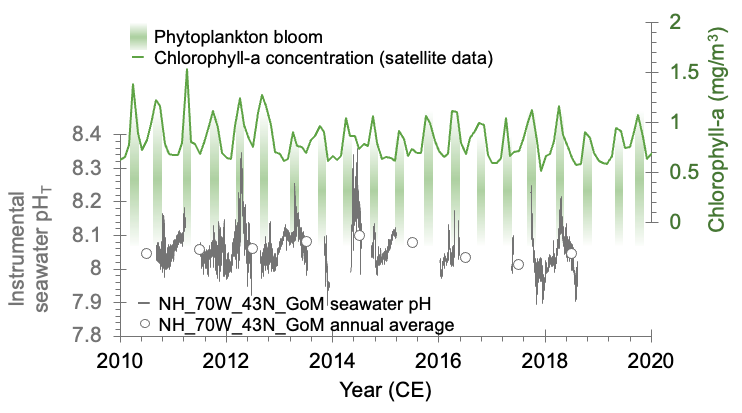


**Supplementary Figure 1**: Sea surface chlorophyll-a concentration as a measure of primary production in the Gulf of Maine from satellite ocean colour data [^1^](#_ENREF_1) compared to recent instrumental surface seawater pH data the NH_70W_43N_GoM monitoring station [^2^](#_ENREF_2). Bi-annual phytoplankton blooms are inferred from chlorophyll-a maxima (green bars). These maxima broadly correspond to seawater pH maxima as surface water dissolved inorganic carbon is removed by primary production.


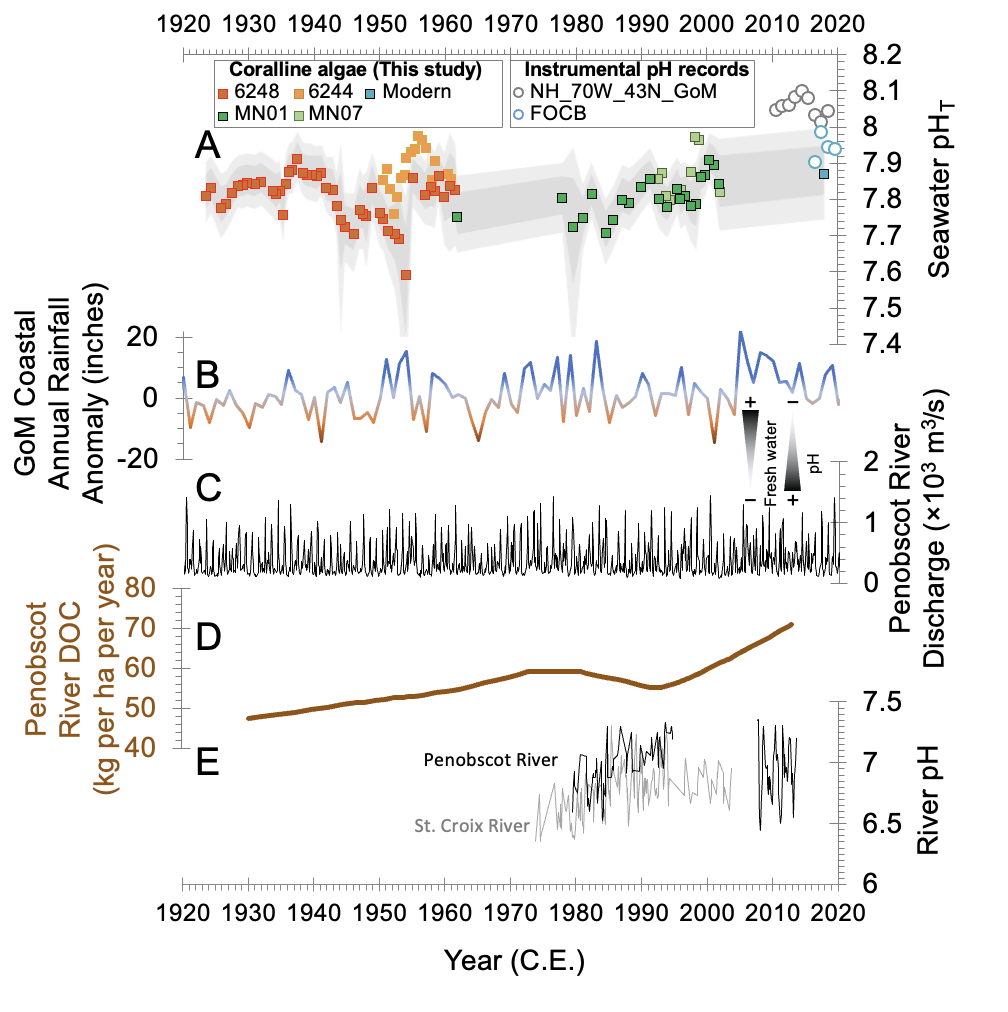


**Supplementary Figure 2**: **A.** Surface water pH (total scale) estimated using *Clathromorphum compactum* coralline algae δ^11^B data and modern instrumental data from Casco Bay (FOCB; [^3^](#_ENREF_3)) and the NH_70W_43N_GoM monitoring station [^2^](#_ENREF_2). **B.** Annual rainfall anomaly for the coastal Gulf of Maine region [^4^](#_ENREF_4). **C.** Penobscot River discharge measured at West Enfield, Maine [^5^](#_ENREF_5). **D.** Modelled Penobscot River dissolved organic carbon (DOC) export flux [^6^](#_ENREF_6). **D.** River water pH measured in Penobscot River (Eddington, Maine) and St. Croix River (Milltown, Maine; ~150 km North East of study site) [^5^](#_ENREF_5).


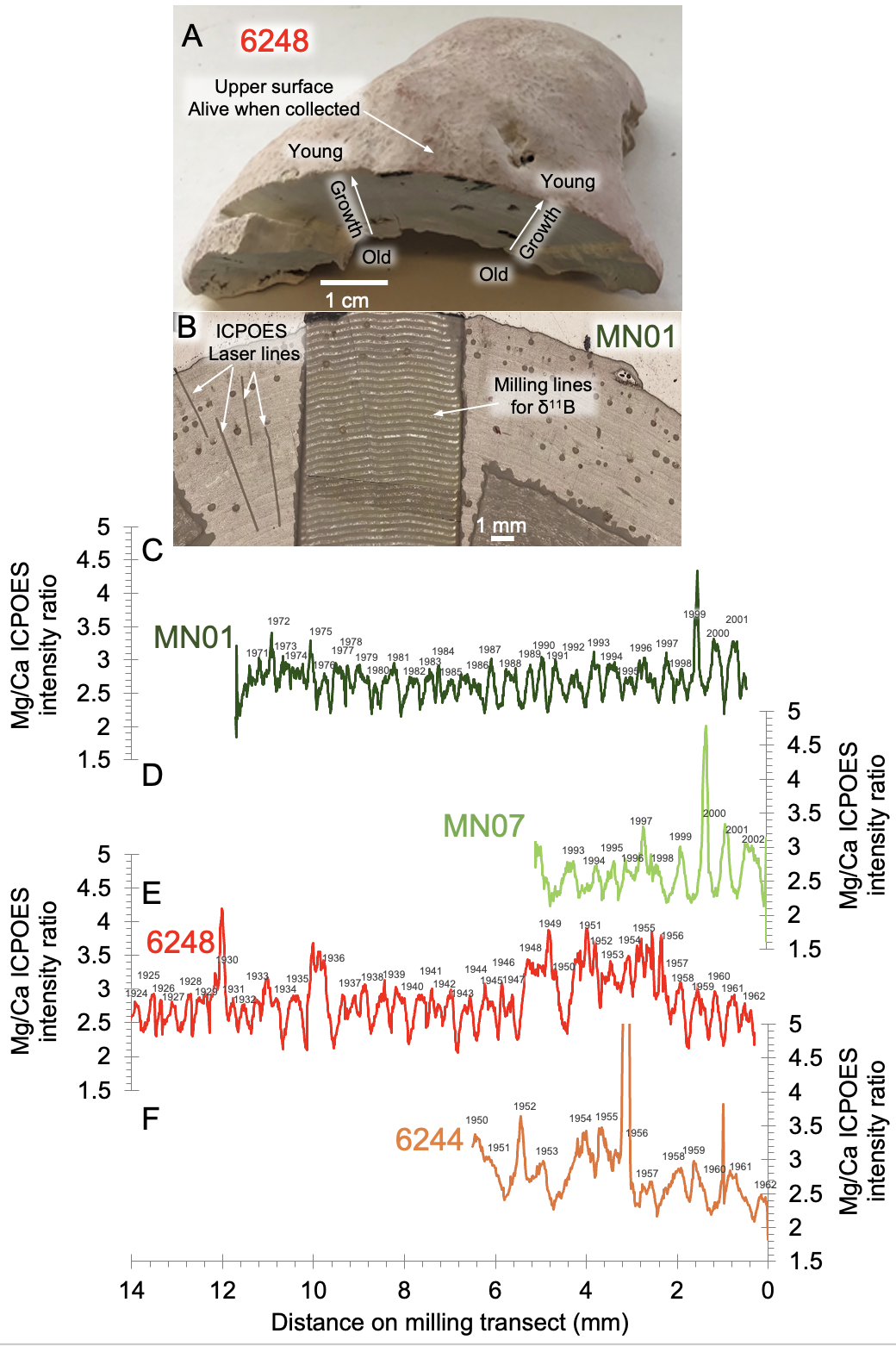


**Supplementary Figure 3**: Growth band dating of *C. compactum* coralline algae samples. (**A**) Sectioned sample 6248. (**B**) Polished cross section of specimen MN01 with micromill paths and ICP-OES transect lines measured for Mg/Ca. (**C - F**) Seasonal cycles in Mg/Ca (high temperature = high Mg/Ca) across growth of a coralline algae specimens and their calendar year assignment. These Mg/Ca cycles are used to assign the age of the samples in conjunction with visual growth band counting (e.g. [^7^](#_ENREF_7)).


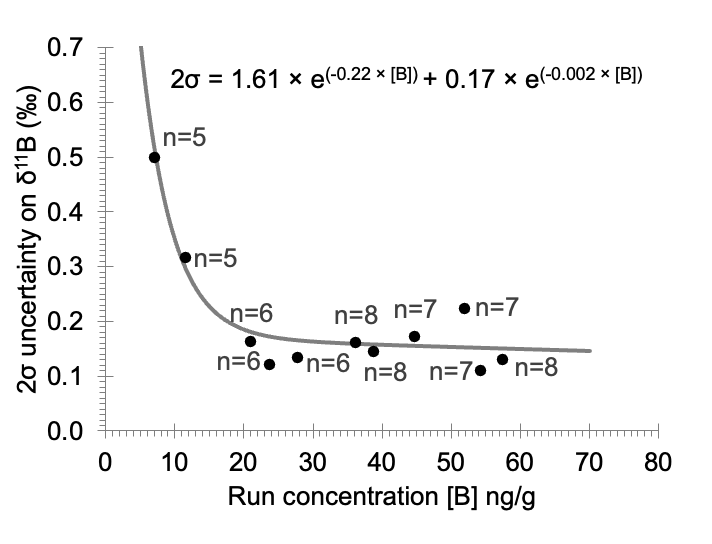


**Supplementary Figure 4**: δ^11^B measurement uncertainty dependency on [B] of the sample solution. Reproducibility (2σ) is assessed using repeat measurements (n) of NIST RM 8301 (Coral) dissolved carbonate reference material at varying boron concentrations. A double exponential fit is applied to the results following the protocol of [Rae, et al. ^8^](#_ENREF_8) (MATLAB curve fitting tool).


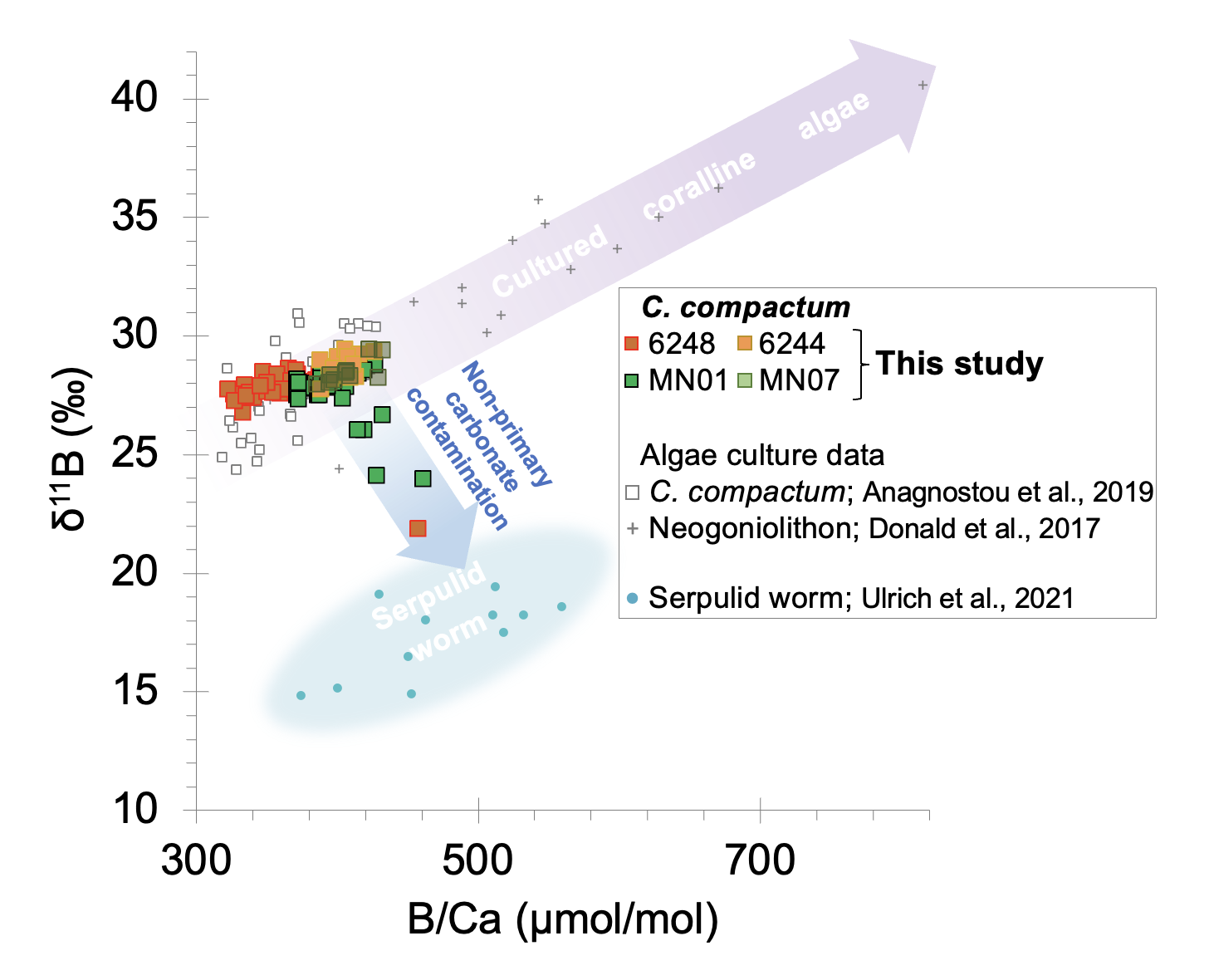


**Supplementary Figure 5**: Screening for contamination. Coralline algal B/Ca and δ^11^B show strong positive correlation in *Neogoniolithon* and *Clathromorphum* specimens grown in culture experiments [^9^](#_ENREF_9)^,^[^10^](#_ENREF_10). *Clathromorphum compactum* samples from this study fall on the same positive trend line apart from 7 sub-samples from specimen MN01 and 6248 that potentially show incorporation of non-primary carbonate (blue arrow), likely associated with nearby worm holes in the specimen. These contaminated samples are not included in the pH reconstruction.


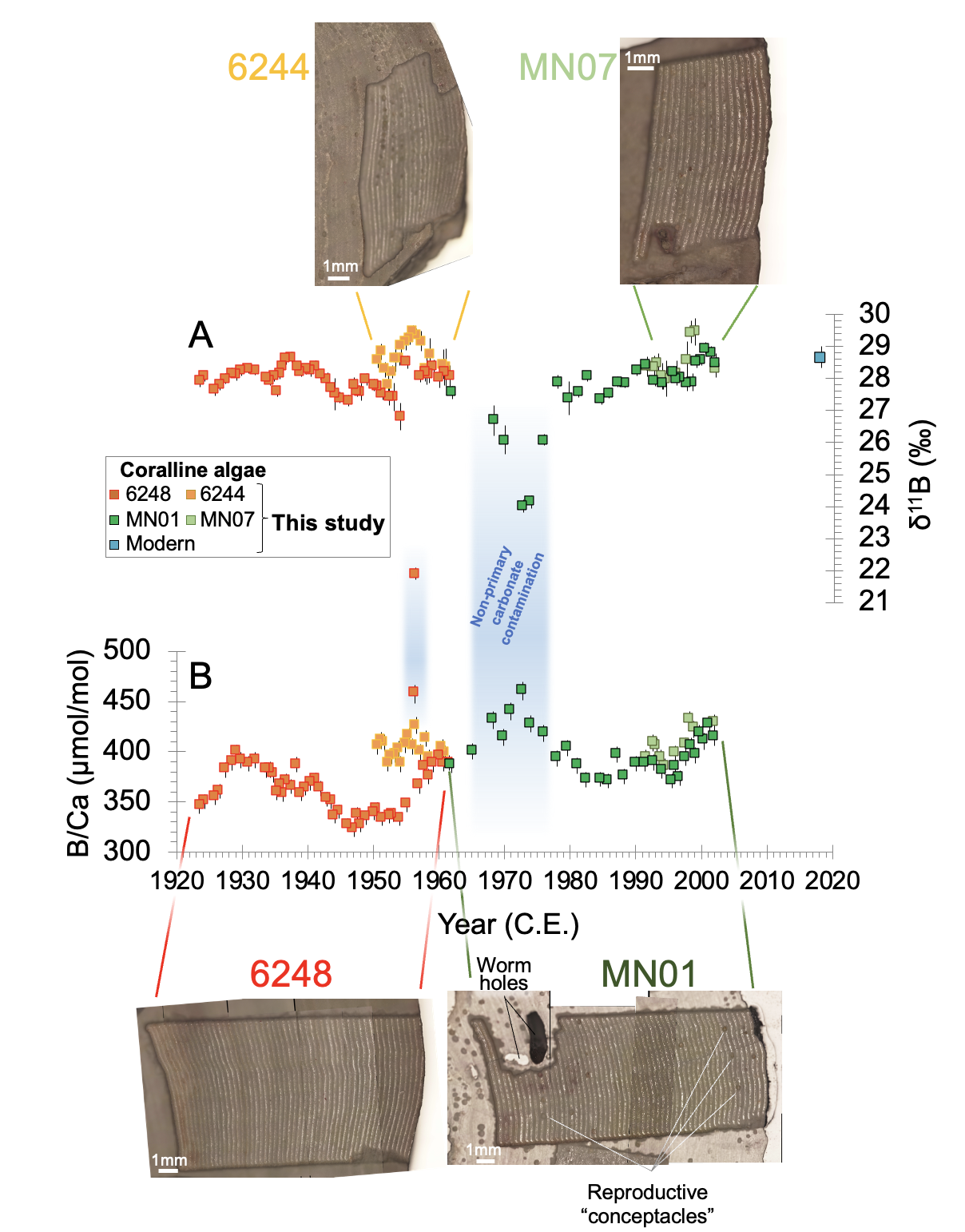


**Supplementary Figure 6**: *C. compactum* coralline algae (**A**) δ^11^B and (**B**) B/Ca data from the Gulf of Maine in this study. Error bars denote analytical uncertainty of 2𝜎 for δ^11^B and 1% RSD for B/Ca. Vertical blue shading denotes interval of altered secondary calcite determined by δ^11^B vs B/Ca correlation in **Supplementary Figure 5**. Sample photographs were taken after micro milling show the growth banding, small reproductive “conceptacle” holes, and larger worm burrows (particularly in MN01).

## References

1 Sathyendranath, S. *et al.* ESA Ocean Colour Climate Change Initiative (Ocean_Colour_cci): Global chlorophyll-a data products gridded on a sinusoidal projection, Version 4.2. Centre for Environmental Data Analysis. <https://catalogue.ceda.ac.uk/uuid/99348189bd33459cbd597a58c30d8d10>. (2020)

2 Sutton, A. J. *et al.* High-resolution ocean and atmosphere pCO2 time-series measurements from mooring NH_70W_43N n the North Atlantic Ocean (NCEI Accession 0115402). (2015)

3 Friends of Casco Bay. *Friends of Casco Bay Water Quality Monitoring Program.* [*www.cascobay.org*](file:///C:\Users\bwilliams\Box\U%20drive\Papers\Manuscripts\Stewart%20d11B%20CCA\Files%20for%20submission\www.cascobay.org), <[www.cascobay.org](file:///C:\Users\bwilliams\Box\U%20drive\Papers\Manuscripts\Stewart%20d11B%20CCA\Files%20for%20submission\www.cascobay.org)> (2020).

4 NOAA National Centers for Environmental Information. Climate at a Glance: Statewide Time Series. <https://www.ncei.noaa.gov/access/monitoring/climate-at-a-glance/statewide/time-series>. (2023).

5 U.S. Geological Survey. Surface Water data for USA: USGS Surface-Water Monthly Statistics. <https://waterdata.usgs.gov/nwis/monthly>? (2023)

6 Huntington, T. G. *et al.* Climate change and dissolved organic carbon export to the Gulf of Maine. *Journal of Geophysical Research: Biogeosciences* **121**, 2700-2716, (2016).

7 Light, T. *et al.* Advancing Mg/Ca Analysis of Coralline Algae as a Climate Proxy by Assessing LA‐ICP‐OES Sampling and Coupled Mg/Ca‐δ18O Analysis. *Geochemistry, Geophysics, Geosystems* **19**, 2876-2894, (2018).

8 Rae, J. W. B., Foster, G. L., Schmidt, D. N. & Elliott, T. Boron isotopes and B/Ca in benthic foraminifera: Proxies for the deep ocean carbonate system. *Earth and Planetary Science Letters* **302**, 403-413, (2011).

9 Anagnostou, E., Williams, B., Westfield, I., Foster, G. L. & Ries, J. B. Calibration of the pH-δ^11^B and temperature-Mg/Li proxies in the long-lived high-latitude crustose coralline red alga Clathromorphum compactum via controlled laboratory experiments. *Geochimica et Cosmochimica Acta* **254**, 142-155, (2019).

10 Donald, H. K., Ries, J. B., Stewart, J. A., Fowell, S. E. & Foster, G. L. Boron isotope sensitivity to seawater pH change in a species of Neogoniolithon coralline red alga. *Geochimica et Cosmochimica Acta* **217**, 240-253, (2017).
